# Supplementary material for: Visfatin-Induced Inhibition of miR-1264 Facilitates PDGF-C Synthesis in Chondrosarcoma Cells and Enhances Endothelial Progenitor Cell Angiogenesis
Source: Cells. 2022 Nov 2;11(21):3470. doi: 10.3390/cells11213470 (PMC9656973; doi:10.3390/cells11213470)

Supplemental data

Materials

p-p85, p-Akt, p-mTOR antibodies were purchased from Santa Cruz Biotechnology (Santa Cruz, CA, USA). Visfatin, PI3K, Akt, mTOR, and β-actin antibodies were obtained from GeneTex (Hsinchu, Taiwan). Rabbit anti-PDGFC (55076-1-AP) antibody was obtained from Proteintech (CA, USA). The siRNAs were acquired from Dharmacon (Lafayette, CO, USA). Cell culture supplements and Lipofectamine 2000 were bought from Invitrogen (Carlsbad, CA, USA). Human chondrosarcoma tissue arrays were purchased from Biomax (Rockville, MD, USA). All other chemicals were purchased from Sigma-Aldrich (St. Louis, MO, USA).

Chorioallantoic membranes (CAM) assay

Specific pathogen-free fertilized chicken eggs were purchased from an animal health research institute (Taipei, Taiwan). The fertilized eggs are incubated in a humid atmosphere at 37 °C and 80%. Under aseptic conditions, a small window was made in the shell on day 3 of chick embryo development. However, Windows were then resealed with adhesive tape, and eggs were returned to the incubator. On day 7, CM from JJ012, JJ012/visfatin, JJ012/visfatin/shPDGF-C, and treat FK866 cells were mixed with Matrigel and deposited in the center of the CAM. The angiogenic response was analyzed 4 days after implantation. Trim the Matrigel plugs from the CAM, collect them for microscopic examination, and take pictures. The number of blood vessels as an index of angiogenesis is quantified by counting the branches of blood vessels. At least 6 viable embryos were tested. All preclinical experiments were conducted according to a protocol approved by China Medical University (Taichung, Taiwan) institutional Animal Care and Use Committees

Matrigel plug assay

Chondrosarcoma cell conditioned medium (CM) was collected from JJ012, JJ012/visfatin, JJ012/visfatin/shPDGF-C, and JJ012/visaftin+FK866(100nM). Four-week-old male nude mice were subcutaneously injected with Matrigel containing 0.15 mL chondrosarcoma CM. Matrigel particles were collected 7 days after implantation, fixed in 4% paraformaldehyde/PBS, embedded in paraffin, and stained with CD31. The blood vessel formation of the sample is also quantified by the Drabkin method. All animal experiments were done in accordance with a protocol approved by China Medical University's Institutional Animal Care and Use Committee (Taichung, Taiwan).

***In vivo* tumor xenograft model**

Nude mice (4-week of age) were purchased from the National Laboratory Animal Center. All animal experiments were done in accordance with a protocol approved by China Medical University’s Institutional Animal Care and Use Committee (CMUIACUC-2019-079). All mice were housed in the Animal Center of China Medical University (Taichung,

Taiwan) and were adapted to the environment one week before the experiment. Four-week-old male BALB/c-nu mice were used for allocating treatment. The exponentially growing culture was resuspended in 200 μl 1.5×10^6 cells JJ012, JJ012/visfatin and JJ012/visfatin/shPDGF-C containing 50% serum-free DMEM/α-MEM and 50% Matrigel. It is transplanted subcutaneously to the right side. Experimental JJ012/visfatin groups were intraperitoneally injected every two days for 15 days with 25ng/g FK866 (visfatin inhibitor). After 4 weeks, the mice were sacrificed by CO_2 inhalation and the tumors were removed, and fixed in 10% formalin, and then the tumor volume and weight were measured.

Immunohistochemistry (IHC) Staining

The human chondrosarcoma tissue array OS802c was purchased from US Biomax Inc. (MD, USA). Human or mouse tissue sections were deparaffinized with xylene and rehydrated by adding ethanol. Endogenous peroxidase activity was blocked with 3% hydrogen peroxide in methanol for 10 min. Heat-induced antigen retrieval was carried out for all sections in 0.01 M sodium citrate buffer, pH 6 at 95°C for 25 min. Human PDGF-C antibodies were applied at a dilution of 1:200 and incubated at 4°C overnight. The antibody-binding signal was detected using the NovoLink Polymer Detection System (Leica Microsystems) and visualized with the diaminobenzidine reaction. The sections were counterstained with hematoxylin and eosin (H&E) and scored according to the extent of the staining: 0, no staining; 1, <10% of cells stained; 2, 10–25% of cells stained; 3, 25–50% of cells stained; 4, 50–75% of cells stained; 5, >75% of cells stained. Using a Computing Densitometer and ImageQuant software (Amersham Biosciences), the final immunoreactive score was determined by multiplying the intensity and extent of positivity scores of stained cells.

Figure S1. EPCs were incubated with visfatin for 24 h. The cell proliferation was examined by MTT assay.

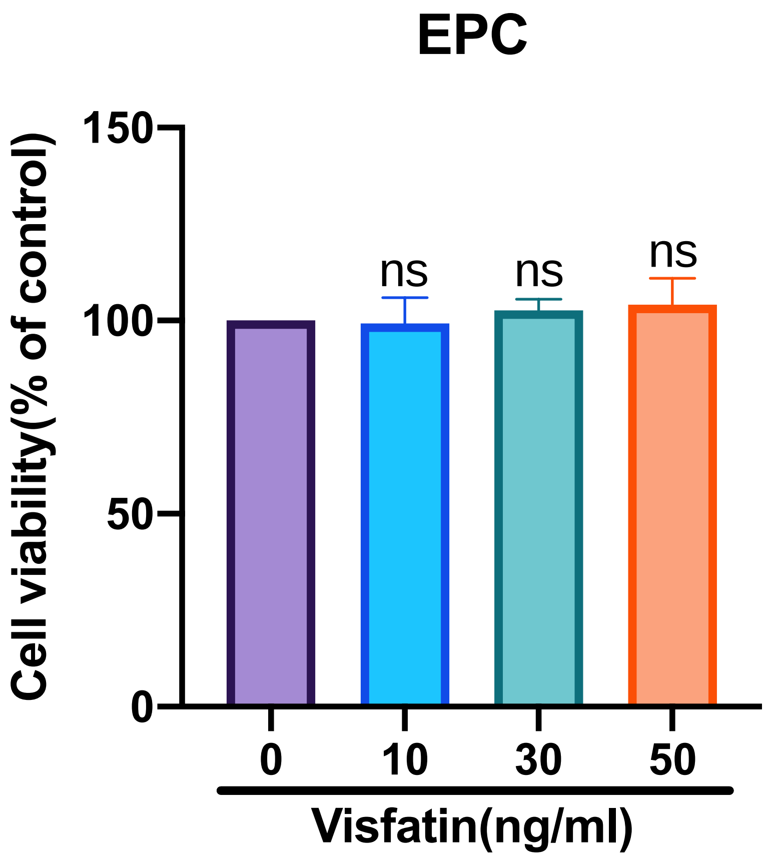

Figure S2. EPCs were incubated with the indicated CMs, then photographed under a microscope.

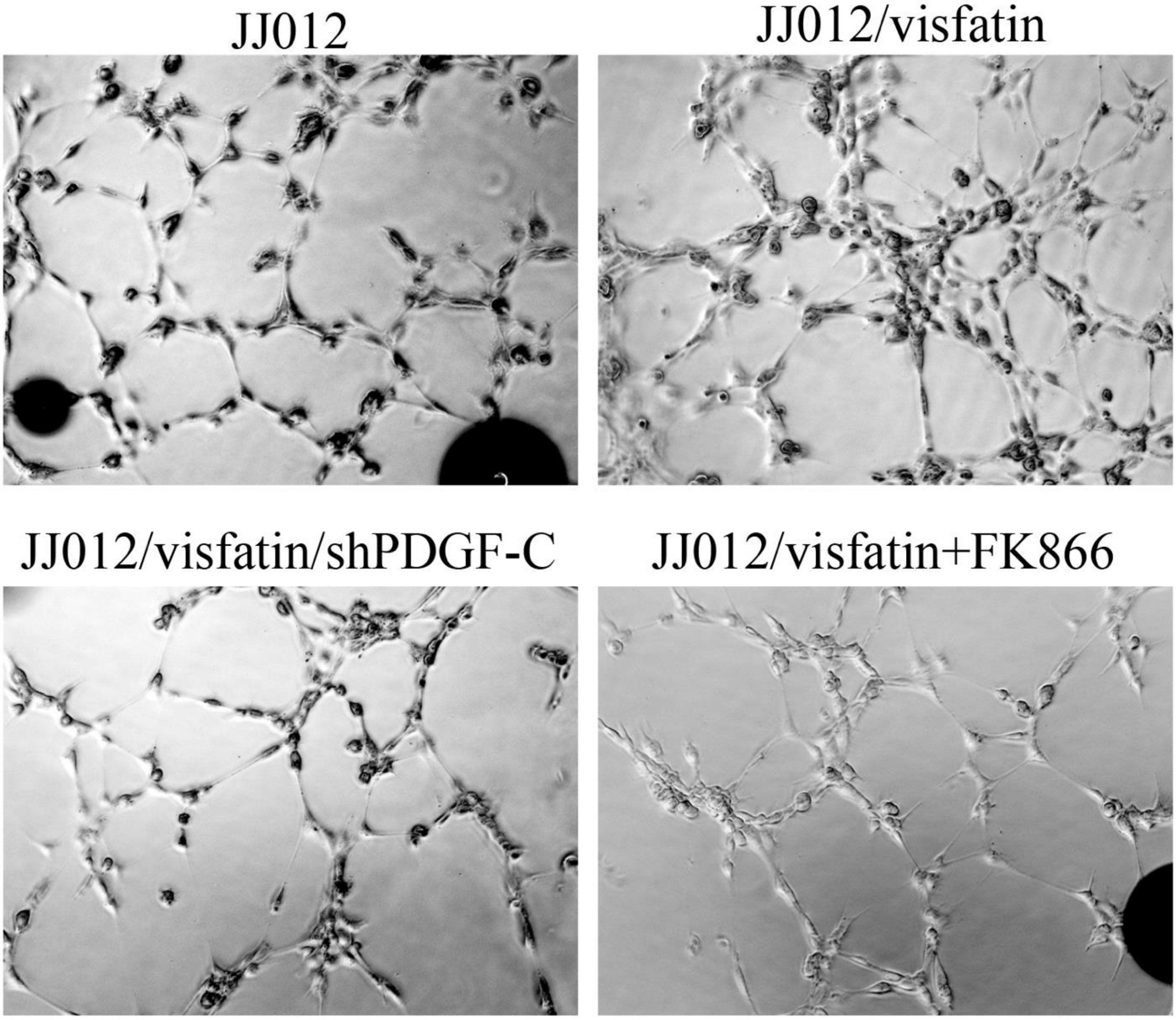

Figure S3. JJ012 cells were incubated with visfatin for 24 h and miRNAs expression was examined by RT-qPCR. The top five low-expression candidate miRNA, including (a) miR-1264, (b) miR-29a-3p, (c) miR-9-5p, (d)miR-548-3p, and (e) miR-944 were treated with visfatin in different concentrations. * $P < 0.05$ compared with the control group.

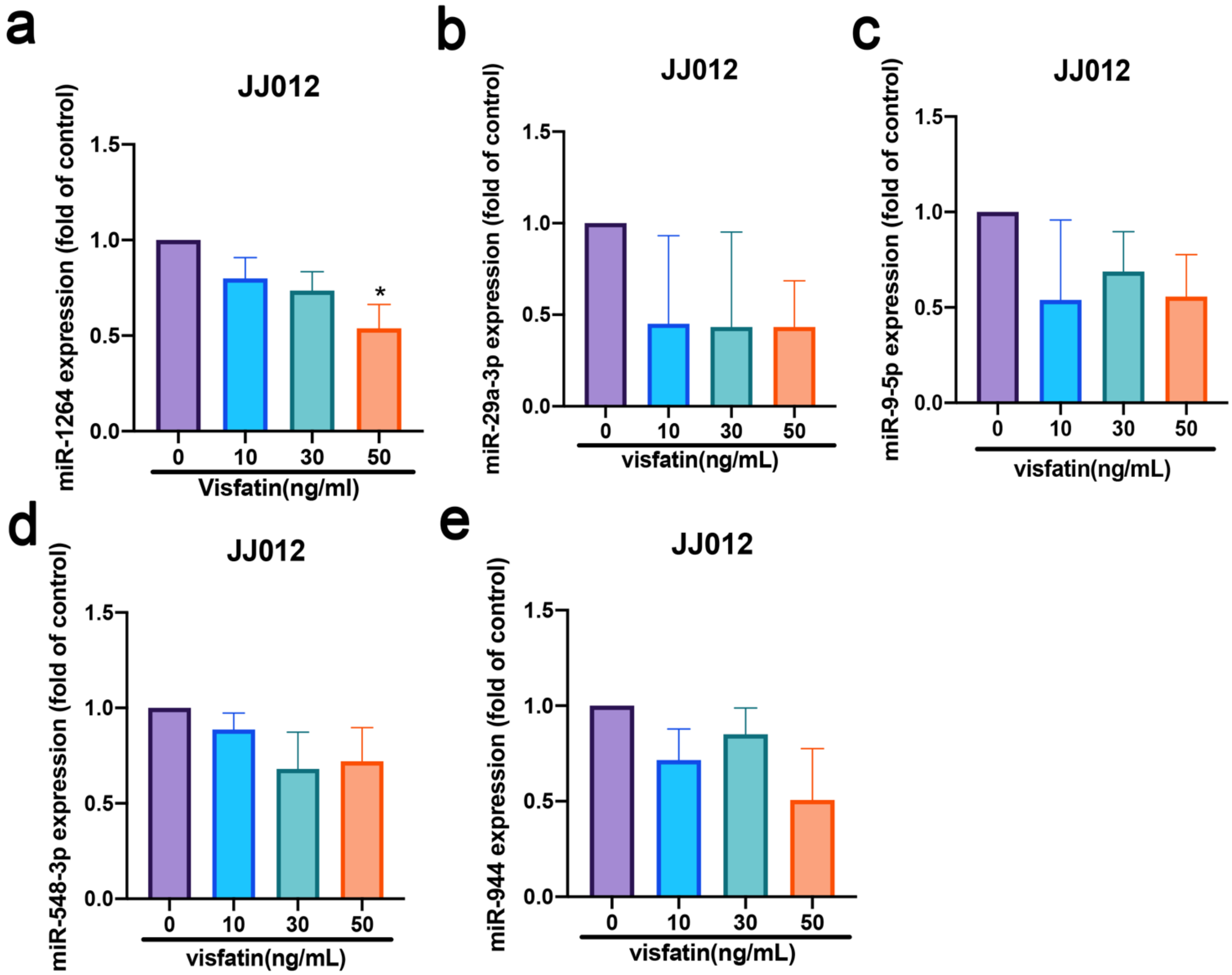

Figure S4. JJ012 cells were pretreated with ERK, p38, JNK, FAK and PKCδ inhibitor for 30 min then stimulated with visfatin for 24 h. RT-qPCR determined PDGF-C expression.

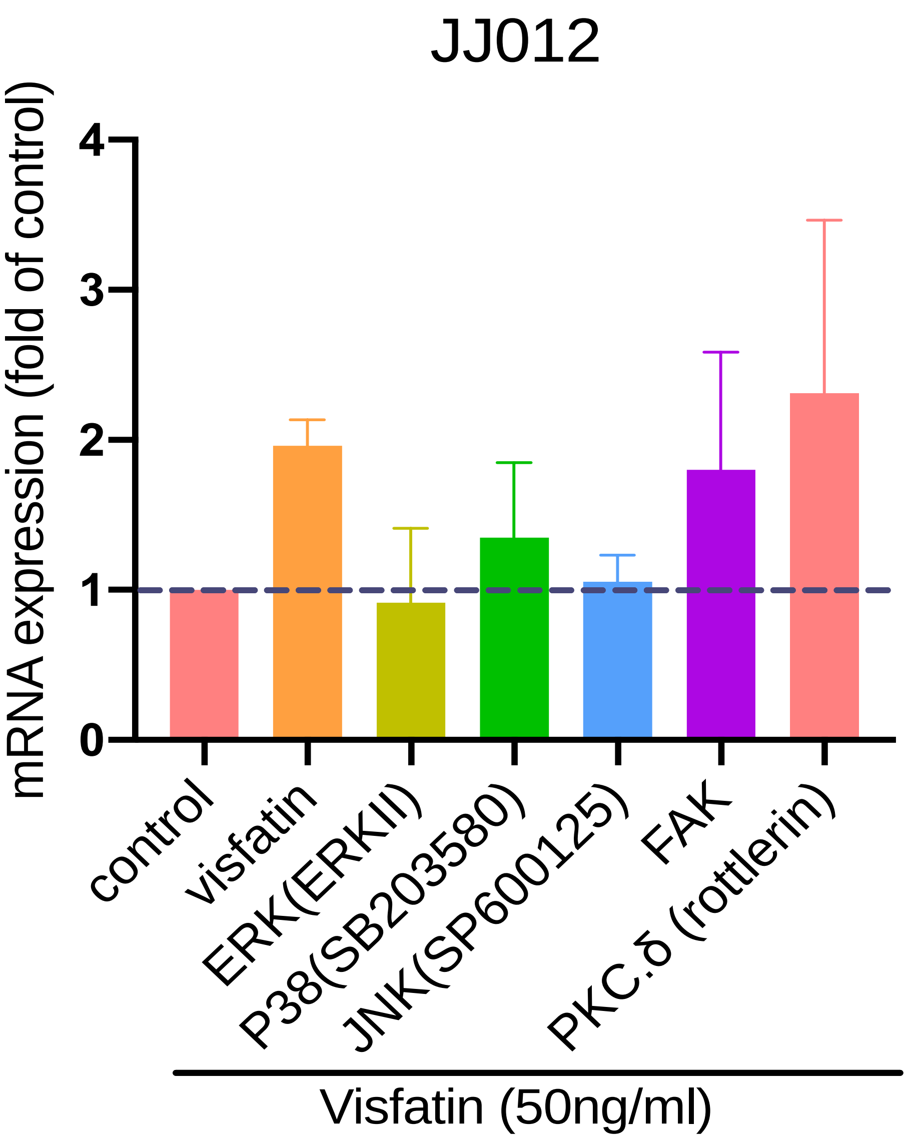

Supplement: Supplementary file 1 [file cells-11-03470-s001.zip › cells-1962070-supplementary.pdf]
